# What Do Spanish Speakers Think of the Andar, Hablar, Ojos, Rostro, Ambos Brazos o Piernas (AHORA) Stroke Tool?

**DOI:** 10.7759/cureus.20720

**Published:** 2021-12-26

**Authors:** Trilok Stead, Latha Ganti, Emily McCauley, Helene Koumans, Maricela Wilson, Madison D Weech, Andrew R Barbera, Paul R Banerjee

**Affiliations:** 1 Emergency Medicine, Trinity Preparatory School, Winter Park, USA; 2 Emergency Medicine, Envision Physician Services, Plantation, USA; 3 Emergency Medicine, University of Central Florida College of Medicine, Orlando, USA; 4 Emergency Medicine, Ocala Regional Medical Center, Ocala, USA; 5 Emergency Medicine, Hospital Corporation of America (HCA) Healthcare Graduate Medical Education Consortium, Olrando, USA; 6 Emergency Medicine, Brown University, Providence, USA; 7 Stroke Community Outreach, Seton Healthcare Family, Austin, USA; 8 Biology, University of Florida, Gainesville, USA; 9 Department of Emergency Medicine, Lakeland Regional Health, Lakeland, USA; 10 Emergency Medical Services, Polk County Fire Rescue, Bartow, USA

**Keywords:** patient education, health awareness, public health, stroke, ahora stroke scale

## Abstract

AHORA (Andar, Hablar, Ojos, Rostro, Ambos Brazos o Piernas) is a Spanish language tool to identify stroke symptoms. A survey of 300 primarily Spanish-speaking, non-medical professionals was conducted to assess the acceptance of the tool, specifically about ease of understanding and ability to implement it. The overwhelming majority of respondents reacted very positively to the tool, finding it quite easy to learn, teach, and understand. Respondent feedback, pitfalls, and questions for further research are presented.

## Introduction

Every 40 seconds, someone has a stroke in the United States [1]. Stroke is the fifth leading cause of death in the United States and the second leading cause of death worldwide [2,3]. Stroke is an even greater burden amongst the Hispanic population, where it is the third leading cause of death. To emphasize the urgency of stroke treatment and to educate people on various signs of stroke, acronyms such as face-arms-speech-time (FAST), face-arms-stability-talking-eyes-react (FASTER), and balance-eyes-face-arms-speech-time (BEFAST) have been developed and implemented. For people who do not speak English, however, such an acronym is not as helpful. The 2020 US Census estimates that approximately 8.3% of the US population speaks English less than “very well” [4]. The Bureau also notes that the most common language spoken in the US other than English is Spanish [5]. Furthermore, death rates from stroke in largely Spanish-speaking populations have been increasing since 2013 [3]. Therefore, it is reasonable to assume that a large portion of the country that may only be able to speak Spanish is at a major risk for stroke death and that a Spanish-language version of tools such as BEFAST would be beneficial for such populations.

The authors previously developed a new prehospital stroke tool, AHORA (which means “now” in Spanish), to help combat the language barrier and reinforce the necessity to call 9-1-1 as soon as any stroke symptoms are observed [6]. The current study aims to investigate whether primarily Spanish-speaking laypersons could easily understand the tool, teach it, and use it.

## Materials and methods

To assess how the general public feels about the AHORA tool, a survey using a third-party polling service was conducted. The survey research platform uses organic sampling built on random device engagement (RDE) [7]. Using artificial intelligence (AI) to track unique respondent identification, RDE reaches users in their natural environments as they participate in their daily activities through any device. The partnership with over 120,000 applications and more than 700 million global users allows for random recruitment of participants fitting the specific inclusion criteria via in-app incentives specific to each user’s real-time activity on their respective devices [7]. The advanced AI technology and algorithm prevents fraud from single users on multiple accounts and suspicious or illogical responses to specific questions. The survey uses weighting to match the univariate distributions of age, gender, and geographic region. All results are reported using this weighting.

Respondents were specifically chosen to be primary Spanish-speakers and non-healthcare workers to gauge the opinion of the general populace-the group who will likely use this evaluation the most. The respondents were asked a series of questions about their primary language, education level, race, occupation, annual income, previous experience with stroke, gender, and how they felt about the tool’s ability to be taught, understood, and suggestions they had regarding the tool design and content itself. As responses were collected in a de-identified manner, this study was considered exempt by our institutional review board (IRB).

## Results

The median age of the cohort (n=300) was 31 years with an interquartile range of 24-40. Forty-three percent were men. Thirty-six percent were married. Fifty-six percent were parents. The highest educational level achieved was: elementary/middle school (6%), high school (34%), vocational school (13%), university (37%), and postgraduate (9%). A total of 28% had a loved one who had suffered a stroke. Only 58% of the cohort was employed. Of the remaining, 10% were looking for work, 8% were homemakers, 7% were students, and 2% were retired. Sixty-one percent reported low-income status. Almost half of the cohort (45%) were from the Southern United States; 13% were from the Midwest, 16% Northeast, and 25% from the West. In total, 32 states were represented, with Florida, California, and Texas having the highest representation. 

**Figure 1:**
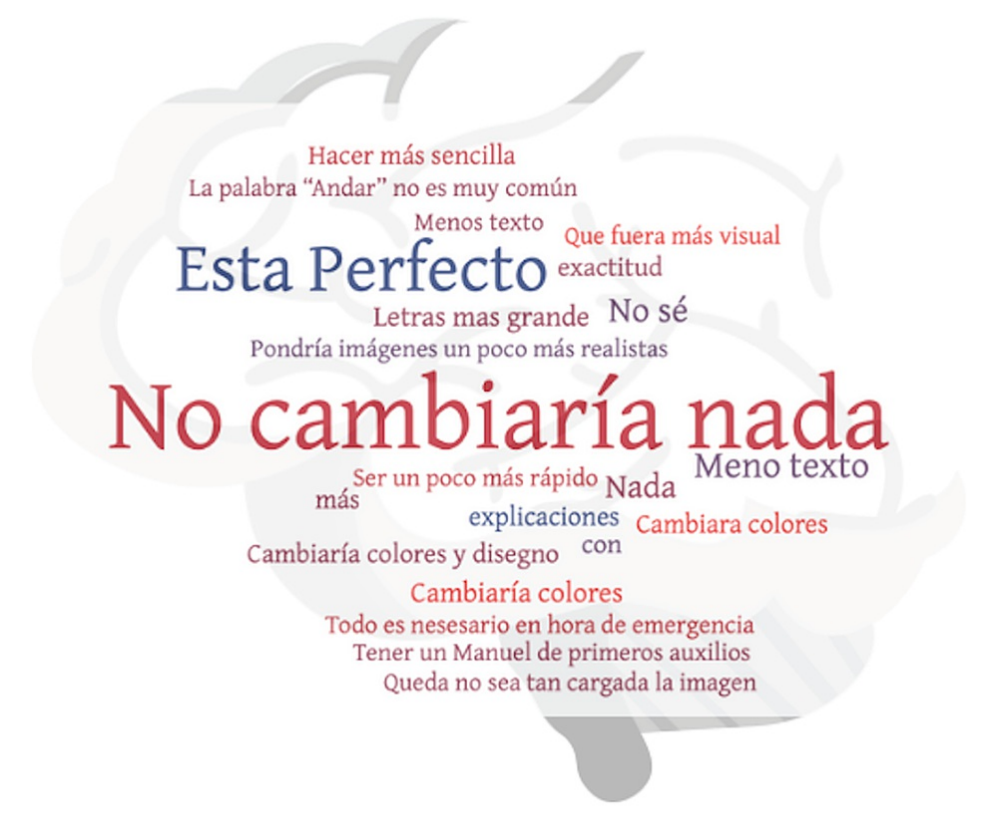
Word-cloud analyses of the major themes among the responses to the question seeking suggestions to improve the AHORA tool.

All respondents noted that the scale was: 1) easy to understand, 2) easy to teach, and 3) easy to implement. When the participants were asked what they would change about the tool, the vast majority (88%) said they would not change anything at all (“no cambiaría nada”), suggesting the tool would likely be effective for this population. Only two respondents suggested additional content, including the phrase “maintain calm” and information on stroke epidemiology. The remainder of the cohort suggested improving the infographic itself. Specific suggestions included changing the red and black color scheme-which was somewhat jarring to the eyes-using more white space, using fewer words, simplifying the infographic, making the letters bigger, and improving the quality of the infographics. The frequency of the suggestions is summarized in a word cloud (Figure 1). The English translations of the original answers as well as the percentages are reported in (Table 1). 

**Table 1:** Original answers, with English translation, counts and percentages

Original answer in Spanish	English translation	Count	Percent
No	No	120	40.00%
No cambiaría nada	Would not change anything	74	24.67%
Esta perfecto	It is perfect	43	14.33%
Menos texto	Less text	14	4.67%
No sé	Don’t' know	14	4.67%
Nada	Nothing	9	3.00%
Cambiaría colores	Would change the colors	6	2.00%
Letras mas grande	Larger letters	5	1.67%
Cambiaría colores y disegno	Would change the colors and design	2	0.67%
Hacer más sencilla	Make it more simple	2	0.67%
más explicaciones con exactitud	More explanations	2	0.67%
Pondría imágenes un poco más realistas	I would make images a little more realistic	2	0.67%
Que fuera más visual	Make it more visual	2	0.67%
Queda no sea tan cargada la imagen	Make the images less heavy	2	0.67%
Tener un Manuel de primeros auxilios.	Have a first-aid manual	2	0.67%
Todo es nesesario en hora de emergencia	Everything is necessary in time of emergency	2	0.67%
La palabra “Andar” no es muy común	The word "Andar" is not very common	1	0.33%

**Figure 2:**
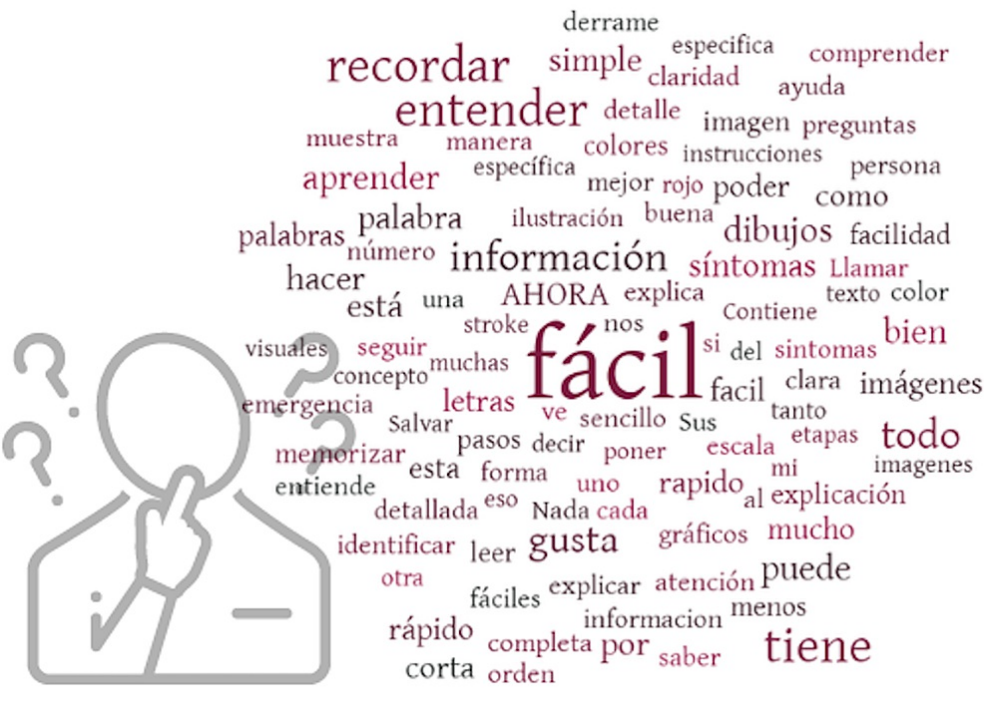
A Word-cloud analyses of the most frequent words in the responses to the question “What do you like most about the AHORA tool?”

When the cohort was asked what they liked about the tool, respondents most frequently noted that it was easy to interpret. Indeed, “fácil” (easy) was the most common word in the word cloud (Figure 2). The English translations of the original answers, as well as the percentages, are reported in Table 2. 

 

**Table 2:** Original answers, English translation, counts and percentages

Original Spanish Phrase	English translation	Count	Percent
es fácil	it is easy	95	31.67%
es fácil de entender	easy to understand	47	15.67%
es fácil de recordar	easy to remember	40	13.33%
es muy facil	it is very easy	27	9.00%
es fácil de aprender	easy to learn	15	5.00%
la información	the information	8	2.67%
los dibujos	the pictures	13	4.33%
los síntomas	the symptoms	7	2.33%
los colores	the colors	7	2.33%
la palabra AHORA	the word AHORA	6	2.00%
la facilidad	the ease	6	2.00%
corta	short	4	1.33%
la explicación	the explanation	4	1.33%
las preguntas	the questions	4	1.33%
bien explicado	well explained	4	1.33%
los pasos	the steps	3	1.00%
corta y fácil	short and easy	2	0.67%
como hacer	how to do it	2	0.67%
el diseño	the design	2	0.67%
las etapas	the stages	2	0.67%
sus gráficos	the graphics	2	0.67%

## Discussion

The main purpose of our simple field study was to assess whether the AHORA tool would be useful for primarily Spanish-speaking persons to recognize the signs and symptoms of acute stroke. This is relevant considering Spanish is the most commonly spoken foreign language in the US. It is estimated to be spoken by 12% (15.2 million) of the US population, with the population growth of Latinos being one of the fastest in the country [8]. Overall, the nationwide cohort was quite satisfied with the AHORA tool, believing it to be quite easy to learn, teach, and understand. Based on these results, adopting AHORA likely has the potential to save countless lives through increased identification of early stroke warning signs and symptoms. Certain changes to the infographic itself will also improve its visual appeal.

According to the 2019 US Census Bureau estimate, there are currently more than 60 million Hispanic individuals living in the USA [9]. This specific population is diverse in many areas, such as language fluency, health insurance status, national origin, and socioeconomic factors. Despite these differences within the Hispanic population, strokes are the third-leading cause of death and the leading cause of disability [10]. In comparison, stroke is the fifth-leading cause of death for the US population as a whole, which highlights the need for improvement in stroke identification and outcomes in the Hispanic population. The American Stroke Association projects that stroke prevalence is expected to increase by 20.5% between 2012 and 2030, with the greatest rise in the subpopulation of Hispanic white men [11]. A contributing factor to these disparities is likely the low adherence to secondary stroke prevention strategies such as compliance with statin or antithrombin medications. This could be in part due to lack of health insurance and below-average health literacy, both of which language barriers could be a major underlying factor. For instance, in a 2021 study of Latino demographics, medications, and vascular risk factors, it was found only 38% of participants with diagnosed atrial fibrillation received anticoagulation. It was also found that lack of health insurance was associated with hyperlipidemia and decreased use of statins [12].

It has been shown that the general population of Mexican Americans are less knowledgeable about stroke risk factors and feel less empowered in preventing stroke compared to the non-Hispanic population [13]. Mexican Americans are less likely to say they would call 9-1-1 when experiencing an acute stroke [10]. This may be improved upon with outreach and increased access to the infographic around communities such as in public buildings, newspapers, medical offices, and schools in areas with a high proportion of Spanish speakers. It has been shown that community-based education can significantly improve stroke knowledge. A study in Washington found a significant increase in stroke knowledge, such as naming symptoms and risk factors for stroke, following a community-based education campaign utilizing television and newspaper ads to inform the public [14]. It can then be expected that utilization of this infographic may have a significant improvement in stroke awareness and education in Spanish-speaking communities.

Important limitations to the current study include those inherent in a survey study. Although the survey utilized weighting to match univariate distributions of age, gender, and geographic region, response bias is inherent in survey research. Respondents who take the time to respond may also be those who have stronger opinions on the topic. There may also have been biased inherent in the phrasing of the question, and certain questions may have been confusing or otherwise provoked some form of bias in the responses.

The next steps include improving the infographic to reflect the suggestions made by the respondents. Additionally, research should be conducted on the AHORA scale to investigate its precision in identifying a stroke. Once validated with such studies, this tool may be utilized in communities across the US, allowing more extensive feedback and subsequent alterations to improve the stroke outcome disparities between populations.

## Conclusions

The AHORA tool appears to be well-regarded among the cohort for learnability, teachability, and comprehension, despite inherent pitfalls in the sampling and questioning. Adopting this tool may help to mitigate the risk of stroke in a demographic that is disadvantaged due to a language barrier, a modifiable risk factor.
